# Using a Low-Power Spiking Continuous Time Neuron (SCTN) for Sound Signal Processing

**DOI:** 10.3390/s21041065

**Published:** 2021-02-04

**Authors:** Moshe Bensimon, Shlomo Greenberg, Moshe Haiut

**Affiliations:** 1 School of Electrical and Computer Engineering, Ben-Gurion University of the Negev, Beersheba 8400711, Israel; bensimmo@post.bgu.ac.il; 2 DSP Group LTD., Herzliya 4659071, Israel; Moshe.Haiut@dspg.com

**Keywords:** spiking neuron, digital neuron, SNN, SCTN, STDP learning rule, LIF model, MFCC, sound feature extraction

## Abstract

This work presents a new approach based on a spiking neural network for sound preprocessing and classification. The proposed approach is biologically inspired by the biological neuron’s characteristic using spiking neurons, and Spike-Timing-Dependent Plasticity (STDP)-based learning rule. We propose a biologically plausible sound classification framework that uses a Spiking Neural Network (SNN) for detecting the embedded frequencies contained within an acoustic signal. This work also demonstrates an efficient hardware implementation of the SNN network based on the low-power Spike Continuous Time Neuron (SCTN). The proposed sound classification framework suggests direct Pulse Density Modulation (PDM) interfacing of the acoustic sensor with the SCTN-based network avoiding the usage of costly digital-to-analog conversions. This paper presents a new connectivity approach applied to Spiking Neuron (SN)-based neural networks. We suggest considering the SCTN neuron as a basic building block in the design of programmable analog electronics circuits. Usually, a neuron is used as a repeated modular element in any neural network structure, and the connectivity between the neurons located at different layers is well defined. Thus, generating a modular Neural Network structure composed of several layers with full or partial connectivity. The proposed approach suggests controlling the behavior of the spiking neurons, and applying smart connectivity to enable the design of simple analog circuits based on SNN. Unlike existing NN-based solutions for which the preprocessing phase is carried out using analog circuits and analog-to-digital conversion, we suggest integrating the preprocessing phase into the network. This approach allows referring to the basic SCTN as an analog module enabling the design of simple analog circuits based on SNN with unique inter-connections between the neurons. The efficiency of the proposed approach is demonstrated by implementing SCTN-based resonators for sound feature extraction and classification. The proposed SCTN-based sound classification approach demonstrates a classification accuracy of 98.73% using the Real-World Computing Partnership (RWCP) database.

## 1. Introduction

In recent years, we are witnessing a shift in technological solutions from the traditional algorithmic approach to multipurpose deep neural networks (DNNs) neuromorphic approach. DNN networks are widely applied to sound recognition and image processing [1,2], presenting a new challenge for low-power and efficient implementation in embedded systems. These types of applications attempt to resemble the operation of the human brain by understanding and imitating the process of human sensory perception. The necessity for low-power and high-performance embedded platforms is increasing [3,4]. Many mobile applications such as the Internet of Things (IoT) are penetrating our life rapidly and require energy-efficient processing units [5], which most of the current NN-based solutions lack. This work presents a new approach based on a spiking neural network for sound preprocessing and classification. The proposed approach is biologically inspired by the biological neuron’s characteristic using spiking neurons, and Spike-Timing-Dependent Plasticity (STDP)-based learning rule. Spiking neural models mimic the biological neurons for information processing purposes [6]. SNN architectures can be used to solve spatiotemporal problems such as in patterns recognition, optimization, and classification problems [7]. SNN have been developed with a neurobiologically plausible computational architecture that incorporates both spatial and temporal data into one unifying model and can be applied for pattern recognition [8]. Recent studies use machine learning methods to integrate the dynamic patterns of spatiotemporal brain data contained in EEG and ERP data. Z. Doborjeh et al. [8] use SNN for evaluation of concurrent neural patterns generated across space and time from electroencephalographic features representing event-related potential (ERP). Unlike classical neurons, a spiking neuron (SN) uses spikes for communication and computations, and therefore has the potential to consume very low power while enabling efficient implementation in terms of silicon area [9]. Bensimon et al. [10] demonstrate that the Spike Continuous Time Neuron (SCTN) model is capable of accurately replicating the behaviors of a biological neuron. A rich diversity of behaviors can be achieved by smart inter-connection of basic neuron blocks. Reordering and connecting blocks of SCTNs into a full SN-based network allow efficient implementation of a large class of cognitive algorithms and voice applications. This work suggests the usage of an innovative energy-efficient hardware-based spiking neuron (SN) presented in [11] as a basic building block in the design of programmable analog electronics circuits.

The proposed SCTN can be considered as a nonlinear device capable of processing an extensive amount of data efficiently. The close resemblance between SN and a biological neuron justifies the evaluation of the substitution of the classical NN model with the SN model. The implementation of SN can be carried out using both analog and digital circuits [12]. Various kinds of SNN-based models and neuromorphic circuits have been recently proposed to support the processing of vast streams of information in real-time [13,14]. A continuous-time spiking neural network paradigm is presented in [15]. The spike latency takes into account that the firing of a given neuron occurs after a continuous-time delay. SNN-based classifier consistently outperforms the traditional Recurrent Neural Network (RNN) and Long Short-Term Memory (LSTM) neural networks in a temporal pattern classification task [16]. Although RNN and LSTM models capture the temporal transition explicitly, they are hard to train for long sound samples due to the vanishing and exploding gradient problem [17]. Low-power spiking-based neural networks are highly suitable to replace RNN and LSTM as the computational engine for time-series applications utilizing the high correlation between adjacent frames [18], especially if the series of data contains strong temporal dependencies like in the case of sound and video applications [14].

STDP is a biologically plausible learning paradigm inspired by the Hebbian learning principle. STDP is one of the most common activity-driven synaptic weight learning mechanisms [19]. Following the STDP learning rule, the neuron weights are adjusted considering the time difference between presynaptic and post-synaptic spikes. According to this rule, if the presynaptic neuron fires earlier than the post-synaptic one, the synapse is strengthened. It has been shown that the unsupervised STDP rule can be used for the detection of frequent input spike patterns [20,21].

This work presents a new building block approach and SCTN-based spiking neural network for sound preprocessing and classification. The proposed approach is biologically inspired by the biological neuron’s characteristic using spiking neurons and STDP-based learning rule. We propose a biologically plausible sound classification framework which uses an SCTN-based network for detecting the embedded frequencies contained within an acoustic signal.

Throughout the years many voice features extraction techniques have been suggested [22,23]. Voice signal identification systems involve the process of converting analog speech waveform into useful features for further processing and classification. Among the leading voice feature extraction techniques are the Mel Frequency Cepstral Coefficient (MFCC) introduced by Davis et al. [24], the Linear Prediction Coefficients (LPC), and the Linear Prediction Cepstral Coefficients (LPCC). The Hidden Markov Modeling (HMM) is one of the most common techniques for voice classification [25] used for automatic speech recognition.

J. Wu et al. present a biologically plausible framework for sound event classification [16]. They propose to use an unsupervised self-organizing maps (SOM) network for representing frequency contents embedded within the acoustic signals and an event-based SNN for pattern classification. R. Xiao et al. propose a feedforward SNN for sound classification using the temporal learning rule [26]. The classification is based on extracting acoustic features from the time-frequency representation of sound (using FFT). They suggest to convert the representative sound features into a spiking train by a simple mapping rather than using SOM. Then these temporal patterns are classified via SNN using temporal learning rules.

Contrarily to the previous SNN-based approaches for sound classification, we propose a framework based on a combined SNN for both preprocessing, feature extraction, and classification. The feature extraction is carried out using an SCTN-based network avoiding the common external preprocessing stage and time-frequency representation of the sound. Moreover, we suggest direct interfacing of the acoustic sensor with the SCTN-based network avoiding the usage of costly analog-to-digital and FFT conversions. This work also demonstrates an efficient hardware implementation of the SNN network based on the low-power digital SCTN neuron presented in [10,11].

The rest of the paper is organized as follows. Section 2 describes in detail the proposed SCTN model and the STDP learning paradigm. Section 3 presents the proposed SCTN building block approach and demonstrates the use of the SCTN to generate a basic frequency detector and a full resonator. Section 4 describes how the SCTN-based SNN is applied to sound features extraction and classification. Section 5 describes the experimental results, and, finally, Section 6 concludes the paper.

## 2. SCTN-Spike Continuous Time Neuron Model

### 2.1. The Leaky Integrate and Fire (LIF) Neuron Model

The proposed Spiking Continuous Timing Neuron (SCTN) is a type of continuous-time representation neuron, whose operation is equivalent to the biological neuron [11]. The SCTN model is based on the common Leaky Integrate and Fire (LIF) model with some modifications allowing various neuron configuration using seven different leak modes and three activation functions with a dynamic threshold setting. The various neuron parameters dictate the type of neuron response to a train of spikes in the time domain. The LIF neuron model is given by Equation (1).

(1) $$Vm_{j}\left(t\right)=Vm_{j}\left(t-1\right)+\sum_{i=1}^{N}w_{ij}\cdot I_{i}\left(t\right)-\mu_{j}$$

where $Vm_{j}\left(t\right)$ is the membrane potential at time *t*, $I_{i}\left(t\right)$ is the binary synaptic inputs (a stream of spikes), and $w_{ij}$ represents the corresponding weights. The weight $w_{ij}$ is the strength of the connection from the *i*th to the *j*th neuron. For each neuron *j*, the index *i* ranges from 1 to *N*, where *N* represents the number of the neurons in the previous layer. The neuron leakage is described by the constant parameter $\mu_{j}$. The proposed LIF neuron model uses a dynamic threshold as an adaptive mechanism to control the firing frequency. When the membrane potential (Vm) exceeds the threshold value, the neuron fires a spike, and then the membrane potential discharges to a resting potential (like a biological neuron) [6]. This default value is determined empirically. The decision to fire and generating a spike in the neuron output is carried out according to the following condition:

(2) $$if\left(Vm\left(t\right)\geq Threshold\right)⟹Spike,Vm\left(t\right)⇐Vm_{reset}$$

**Figure 1:**
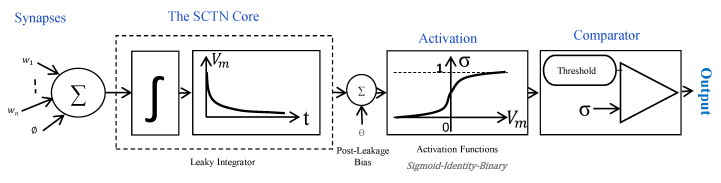
The Spiking Continuous Timing Neuron (SCTN) mathematical model.

Figure 1 illustrates the mathematical model for the proposed SCTN neuron. The proposed spiking neuron structure is composed of four main components: (a) an adder which performs the weighted input summation, (b) a leaky integrator with a time-constant that is controlled by a “memory” parameter α, (c) a random generator which is used to implement the sigmoid activation function, and (d) a comparator that checks if the membrane potential reaches the threshold. The spiking neuron fires a pulse in case the weighted input summed by the adder exceeds the current random value (i.e., threshold).

The SCTN membrane potential, Vm(t), is given by Equation (3).

(3) $$Vm\left(t\right)=\left\{\begin{array}{cc} Vm_{0}, & t=0\\ \left(1-2^{-LF}\right)\left(Vm\left(t-1\right)+\sum_{j=1}^{N}w_{j}\cdot I_{j}+\phi \right)+\Theta , & t\cdot mod(LP)=0\\ Vm\left(t-1\right)+\sum_{j=1}^{N}w_{j}\cdot I_{j}+\phi +\Theta , & t\cdot mod(LP)\neq 0 \end{array}\right.$$

where $t\in N_{0}$, the Leakage Factor (LF), and the Leakage Period (LP) are the neuron leakage parameters representing the integrator leakage rate and the rate of the integrator operation, respectively, and Θ and ϕ are the pre- and post-leakage bias, respectively [10]. The weighted inputs (axons) are accumulated directly into the adder in an iterative process while adding the membrane potential from the previous iteration. The leakage rate is controlled by the α parameter. The membrane potential is represented by a dedicated internal register. A pseudo-random generator is used to generate the nonlinear activation function. In case the accumulated value (including a bias) of the membrane exceeds the threshold, the neuron fires a pulse in its output. The proposed SCTN model consumes less than 10 nW and requires only 700 ASIC 2-inputs gates for implementing a neuron with 10 synapses inputs [10]. The SCTN neuron architecture is comparable, in terms of area and power, to the IBM SN model used in the neuromorphic chip (TrueNorth), which is composed of 1272 gates [27]. Simulations of the proposed digital spiking neuron also demonstrate its ability to accurately replicate the behaviors of a biological neuron model accurately [10].

### 2.2. STDP Learning Module

**Figure 2:**
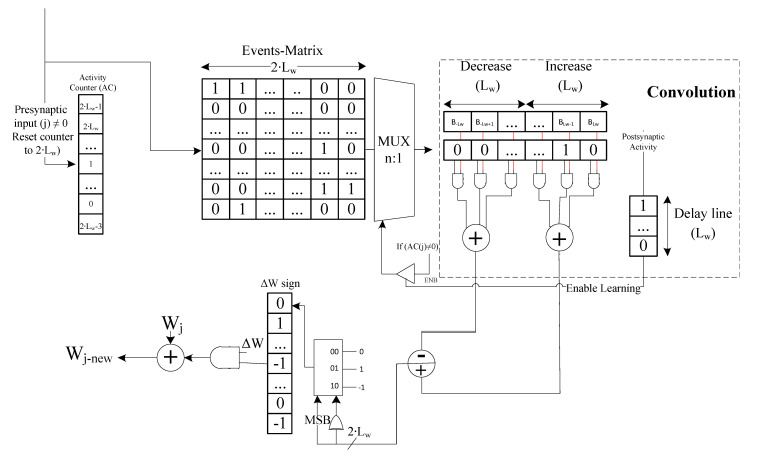
Spike Timing Dependent Plasticity (STDP)-based learning model.

Different types of bio-plausible learning rules are adapted for SNN training [28,29]. These learning rules are based on synaptic plasticity training applying unsupervised learning approaches [30]. Among the most commonly used bio-plausible rules are the Spike Timing Dependent Plasticity (STDP) and the Spike-Driven Synaptic Plasticity (SDSP) [7]. Several hardware STDP implementations have recently been proposed [31,32,33,34]. We adapt the low-power hardware STDP implementation presented in [10] and integrated it in the new SCTN architecture. Figure 2 shows the hardware implementation of the STDP-based learning module. The module is composed of three main components: (a) Time event matrix, (b) STDP weights register, and (c) Post-synaptic delay line. The event-matrix represents the timing of the events, where each row represents an input synapse. Lw is stands for the Length of the considered time Window. The presynaptic spike times are sampled during the time window Lw and stored in an event matrix along with the synaptic index. When the neuron generates a postsynaptic spike, the time of the postsynaptic spike is compared with the stored presynaptic spike times, and the time difference is used to update the weights according to the STDP rule. The time window considers a total of 2Lw time units (pre- and post-firing the spike at the neuron output). Therefore, the event matrix is implemented in hardware using an array of shift registers (one for each input synapse) where each shift-right register is of the length of 2Lw bits. Upon an output spike, the weights of the synapses are updated in relation to the input spike timing and according to the STDP learning rule. Synapses that contribute to the generation of an output spike event should be strengthened. The shifted presynaptic input vector is weighted by Bu which represents the STDP modification function according to the time difference between the postsynaptic presynaptic times spike. The synapse weight update function $W_{j_{new}}$ is built using a convolution of the shifted presynaptic input vector weighted by Bu, where −Lw≤u≤Lw, and the delayed output spike. Different, more complex STDP modification rules can be configured using the proposed B register, eliminating the need for any hardware modification imposed by previous hardware STDP implementation [31]. The STDP learning rule is given by Equation (4).

(4) $$\begin{array}{c} \Delta w_{j}=\eta \sum_{k\in S_{pre}}\sum_{l\in S_{post}}\left\{\begin{array}{cc} +\eta^{+}e^{-|t_{l}-t_{k}|/\tau_{+}}, & if\;t_{l}\leq t_{k}\\ -\eta^{-}e^{-|t_{l}-t_{k}|/\tau_{-}}, & if\;t_{l}>t_{k} \end{array}\right. \end{array}$$

where $\eta^{\left(-/+\right)}$ is the learning rate and $\tau_{+}$ and $\tau_{-}$ are the time scales to control synaptic potentiation and depression. The magnitude of the learning rate decreases exponentially with the absolute value of the timing difference. When multiple spikes are fired, the weight change is the sum of the individual change calculated from all possible spike pairs. $S_{preset}$ and $S_{post}$ are the sets of spikes of the pre- and post-synaptic neurons, respectively, where $t_{k}$ is the time of the spike *k* (of the $S_{preset}$) and $t_{l}$ is the time of the spike *l* (of the $S_{post}$ set).

## 3. Building Block Approach

This section presents the proposed building block approach applied to sound feature extraction and classification. We suggest considering the Spike Continuous Time Neuron (SCTN) presented by Bensimon et al. [10] as a basic “building block” in the design of programmable digital electronics circuits. The proposed approach demonstrates the use of the SCTN neuron as an efficient alternative to the design of programmable analog electronics circuits. The SCTN is used as the basic building block to implement the analog-like circuits, utilizing the unique features of the SCTN neuron and smart inter-connections. Usually, a neuron is used as a repeated modular element in any neural network structure, and the connectivity between the neurons located at different layers is well defined. Thus, generating a modular NN structure composed of several layers with full or partial connectivity. The proposed approach suggests controlling the specific behavior of each neuron and uses smart and unique connectivity of each neuron in order to emulate an analog circuit. Unlike existing SNN-based sound classification solutions for which the preprocessing phase is carried out using analog-to-digital and FFT conversions of the raw analog data, we suggest integrating the preprocessing phase into the network. This approach allows referring to the basic SCTN neuron as an analog module (i.e., a building block), and thus enabling the design of simple analog circuits based on SNN with unique inter-connections between the neurons. The proposed approach is applied to sound signal processing demonstrating efficient ultra-low-power implementation of some common analog circuits used for phase detection and sound feature extraction and classification. The benefit of using an SNN-based network for implementing analog circuits is reflected by a generic solution and the reuse ability by changing the network weights. The building block approach is applied to sound signal processing demonstrating efficient ultra-low-power implementation of some common analog circuits used for phase detection, frequency detection, and sound feature extraction and classification.

### 3.1. SCTN-Based Phase Shifting

**Figure 3:**
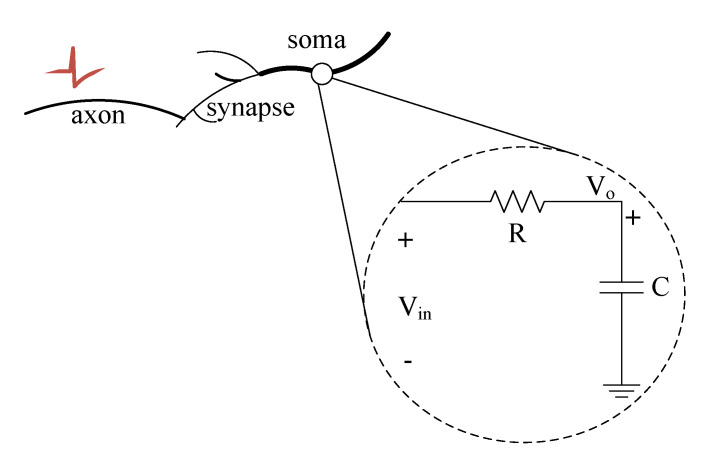
RC circuit filter and its relation/analogy to the SCTN.

The efficiency of the proposed approach is demonstrated by implementing an SNN-based preprocessing phase-shifting circuit and frequency detection. Therefore, enabling a simple and direct sensor interface, saving silicon area, and efficient low-power sound processing in real-time. We examine and demonstrate the ability of the SCTN neuron to generate a phase-shifting emulating a common analog filter circuit. Figure 3 shows a simple first-order RC circuit.

The transfer function of the RC circuit is given by

(5) $$H\left(j\omega \right)=\frac{V_{o}}{V_{in}}=\frac{1-j\omega RC}{1+{\left(\omega RC\right)}^{2}}$$

where the radial frequency is ω=2πf. For the cutoff radial frequency $\omega_{0}=\frac{1}{RC}$ the magnitude and phase are given by Equations (6) and (7).

(6) $$H\left(j\omega_{0}\right)|=\frac{1}{\sqrt{2}}$$

(7) $$arg\left(H\left(j\omega_{0}\right)\right)=\frac{-\pi }{4}$$

Therefore, for an input frequency of $\omega_{0}$, a phase shift of $-45^{\circ }$ is observed. In analogy with an SN neuron, the charge of the capacitor *C* may represent the neuron membrane potential, while the resistor *R* may represent the leakage rate of the neuron [35]. Each SCTN cell incorporates a leaky integrator with a time constant that is controlled by a predefined parameter: $\alpha =1-2^{-LF}$. The SCTN may postpone the incoming signals according to an expected leakage time constant, i.e., a delay which is given by Equation (8).

(8) $$Delay=T_{PulseCycle}\cdot \frac{1}{1-\alpha }\cdot \left(a+LP\right)$$

where $T_{PulseCycle}$ is derived from the system clock as

(9) $$T_{PulseCycle}=\frac{x+1}{2}\cdot \left(T_{sys=clk}\right),-1\leq x<1$$

The SCTN delay and the spike rate are derived from Equation (8) and formulated as

(10) $$\tau =T_{Pulses}\cdot \frac{1}{1-\alpha }\cdot \left(1+LP\right)$$

(11) $$\omega_{0}=\frac{1-\alpha }{T_{Pulses}\cdot \left(1+LP\right)}$$

(12) $$f_{Pulses}=\frac{1}{T_{Pulses}}$$

where τ denotes the delay, and the analog cutoff frequency $f_{0}$ is given by

(13) $$f_{0}=\frac{f_{pulses}\cdot \left(1-\alpha \right)}{2\pi \cdot (1+LP)}=\frac{f_{pulses}}{2^{LF}\cdot 2\pi \cdot \left(1+LP\right)}$$

Therefore, for an appropriate choice of the LP and LF parameters (which determine the resonance frequency), the SCTN has the ability to generate a phase shift of 45 degrees.

### 3.2. SCTN-Based Phase Shifting

This section presents an SCTN-based resonator circuit which serves as a frequency detection utilizing the SCTN basic phase shifting feature. The proposed resonator is used to extract the representing frequency contents embedded within the acoustic signals.

**Figure 4:**
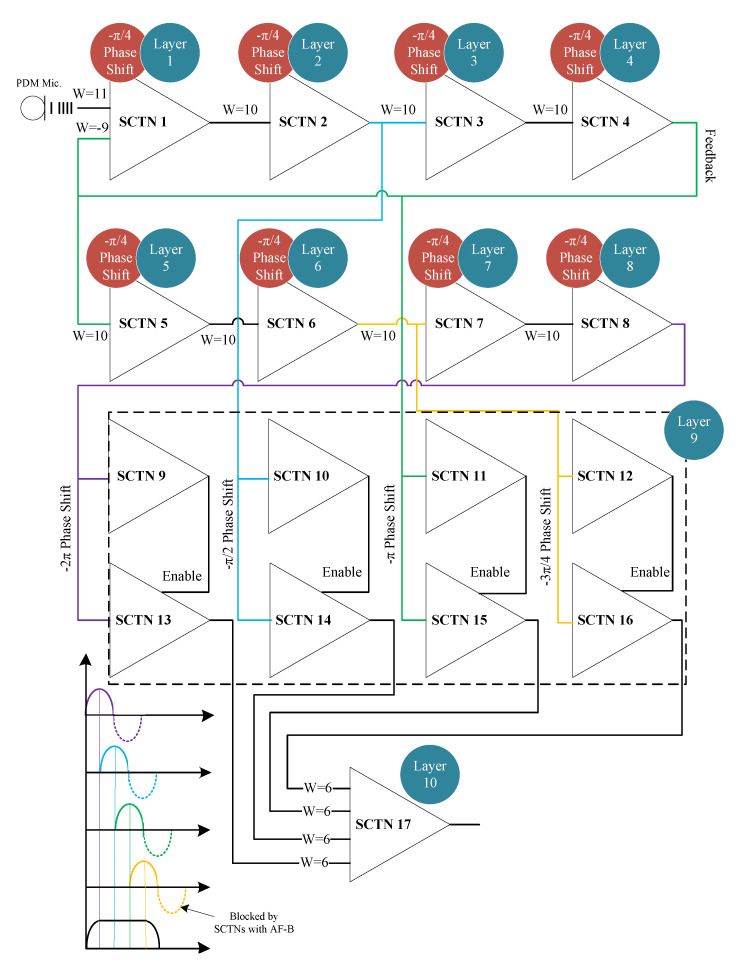
The proposed 10-layer resonator architecture.

Figure 4 depicts a unique SCTN-based resonator circuit. The resonator circuit is used to detect the presence of a range of frequencies embedded within the sound signal. The proposed resonator circuit is composed of 17 SCTNs building blocks arranged in 10 layers. A PDM binary stream representing the sound signal serves as an input to the resonator circuit. The first eight layers are composed of eight SCTN neurons (one per layer), where each neuron performs a phase shift of 45 degrees. A feedback path (connected to the 4th neuron) representing a shift of 180 degrees is connected to the input. The final layer contains 8 SCTN neurons: the first four neurons are used for blocking negative signals, while the other four serves as rectifiers configured with binary and identity activation functions, respectively. In the final stage, the output SCTN neuron sums the four positive outputs of each detected phase (0–90–180 and 270 degrees). The output neuron fires a train of pulses as a result of detecting frequencies that are in the range of the resonance frequency (in a predefined range of frequencies). The maximum pulse rate is achieved at the resonance frequency, and the pulse rate is relatively decreased as the frequency moves away from the resonant mid-frequency.

**Figure 5:**
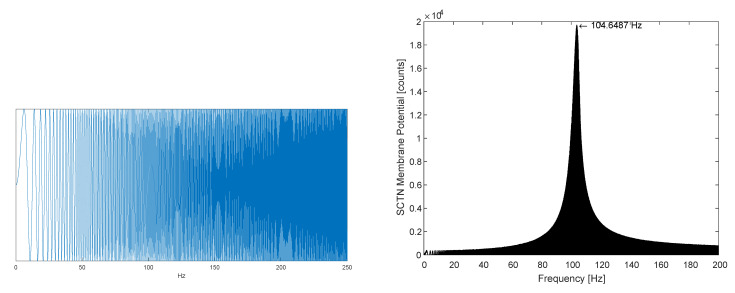
(**left**) Resonator input: a chirp signal. (**right**) SCTN-based Resonator output: detected frequency.

Figure 5 demonstrates the detection of a predefined resonance frequency within a chirp signal containing frequencies in the range of 0 to 250 Hz. The SCTN parameters LP and LF are configured to detect a resonance frequency of $f_{0}=104.65$ [Hz] using Equation (14) for LF = 5, LP = 73, and system clock Fpulse = 1.536 MHz.

(14) $$f_{0}=\frac{1.536\cdot 10^{6}}{2^{5}\cdot 2\pi \cdot \left(1+73\right)}=104.65$$

Figure 5(right) shows that the peak is achieved exactly at the resonance frequency $f_{0}$.

In order to detect several frequencies, which represent the sound signal, within a predefined range, we suggest using a bank of filters (i.e., bank of resonators). Each resonator is responsible for detecting a single frequency. For example, to detect 20 predefined frequencies in the range of [20 Hz, 20 kHz], a bank of 20 filters (composed of 17×20 = 340 SCTN neurons) is required.

**Figure 6:**
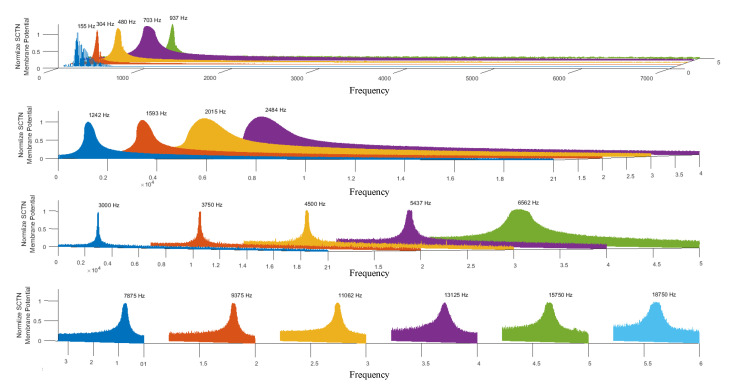
SCTN-based resonators’ bank (the parameters are tuned to detect 20 predefined frequencies in the audio spectrum).

Figure 6 illustrates the simulation results for a bank of 20 resonators (based on SCTN) used to detect 20 different frequencies in the audio spectrum. A chirp signal (in the range of 20 to 20k Hz) has been used as an input to each of the 20 resonators. The array of resonators was tuned to detect the following frequencies: [155; 304; 480; 703; 937; 1242; 1593; 2015; 2484; 3000; 3750; 4500; 5437; 6562; 7875; 9375; 11,062; 13,125; 15,750; 18,750].

This approach can be easily applied for extracting the common MFCC coefficients representing a sound signal. Each resonator can be configured to detect a relatively closed neighborhood of frequencies around the central detected frequency. The bank of filters can be adapted to identify different types of soundtracks (e.g., female and male, background, noises, etc.).

## 4. SCTN-Based Sound Feature Extraction

### 4.1. Classical Sound Preprocessing

**Figure 7:**
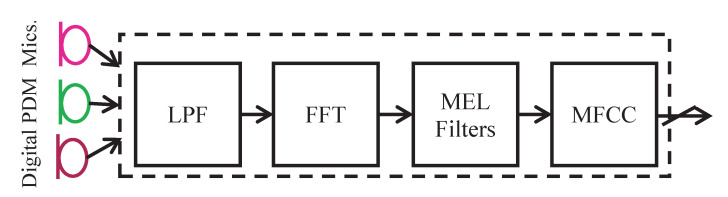
Classical sound preprocessing approach.

Traditionally, a complete sound processing framework consists of the following main stages: Pulse Density Modulation (PDM), preprocessing, features extraction, and classification. MFCC is the most widely used feature extraction method for automatic speech recognition. Figure 7 depicts the stages of classical sound preprocessing and features extraction. The digital microphones translate their vibration intensity from an audio wave to digital information. The PDM signal is converted to Pulse Code Modulation (PCM) representation by a digital Low-Pass Filter (LPF) with downsampling to a sampling rate that is appropriate to voice processing (typically 3–16 KHz). The PCM samples are then buffered into 20 [ms] frames and converted to the frequency domain via FFT transform. The extraction of the MFCC coefficients is achieved by multiplying (in the frequency domain) the Mel filters [24] and the spectral transform of the signal. This roughly determines the energy distribution in the frequency domain. Usually, the sound preprocessing stage is implemented using analog hardware [36].

### 4.2. SCTN-Based Resonators Applied to Features Extraction

**Figure 8:**
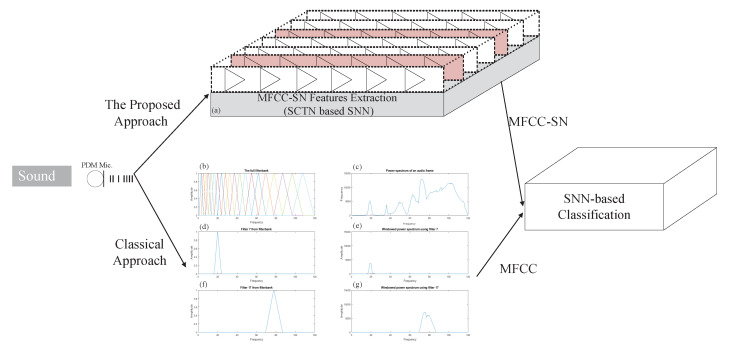
(**a**) SCTN-based features extraction. (**b**–**g**) Classical Mel Frequency Cepstral Coefficient (MFCC) extraction.

The usage of the SCTN-based resonator for frequency detection can be extended to extract features representing a sound signal. Each resonator can be reconfigured to detect a relatively closed neighborhood of frequencies around the central detected frequency. The proposed approach disassembles the sound signal into its fundamental spectral components by detecting the frequencies presented in the audio signal. Figure 8 depicts the classical approach for MFCC extraction along with the proposed SCTN-based feature extraction (SN-MFCC).

Figure 8a shows the proposed SCTN resonator-based filter banks used to extract the SN-MFCC features, while Figure 8b–g demonstrate the classical MFCC extraction. Figure 8b describes the MEL filters; Figure 8c depicts the FFT domain of a typical sound frame. Figure 8e,g depicts the multiplication of two representative MEL filters shown in Figure 8d,f by the spectral transform of the signal in Figure 8c. The extracted MFCC features are used for sound classification using an SCTN-based classifier, although the extracted SN-MFCC coefficients do not precisely match the classical MFCC.

The SCTN-based classifier is composed of one layer with a dedicated SCTN neuron per class. The SCTN-based classifier has been trained separately for each class using the STDP learning rule. The classical MFCC coefficients should be extracted iteratively in real-time for each sampling frame (typically 20 ms frames with 10 ms overlap). By contrast, the proposed approach suggests processing the audio signal continuously, avoiding the need to split the input signal into frames. The input data can be connected directly to a digital PDM microphones that produces rate coded spikes. Moreover, in contrast to the frame-based system, as the SCTN-based preprocessing is driven by an external stimulus, unnecessary continuous frame processing (like silence frames) is avoided.

## 5. Experimental and Results

The proposed sound classification based on the SN-MFCC extracted features was evaluated using the Real-World Computing Partnership (RWCP) standard database [37]. To allow fair comparisons with other existing SNN-based sound classification systems [16,26] we select the same ten classes from the RWCP database (‘cymbals’, ‘horn’, ‘phone4’, ‘bells5’, ‘kara’, ‘bottle1’, ‘buzzer’, ‘metal15’, ‘whistle1’, and ‘ring’). The sound files are recorded at 16 kHz sampling rate in a noisy background. The SCTN-based classification network was trained and tested with 40 randomly selected sound samples per class, where 20 samples have been used for training and 20 samples used for testing. To evaluate the generalization abilities of the proposed SCTN-based classifier the test data set includes only new different sound samples. The test data set contains 200 randomly selected sound samples. A cross-validation, using different randomly partitioning of the dataset for training and testing, is carried out to test the ability of the proposed approach to correctly classify new data that was not used in the training process. The training and testing process was repeated 10 times with different sound samples randomly selected from the RWCP database. To reduce variability, multiple rounds of cross-validation are performed using different dataset partitions, and the validation results are averaged over the 10 rounds.

**Figure 9:**
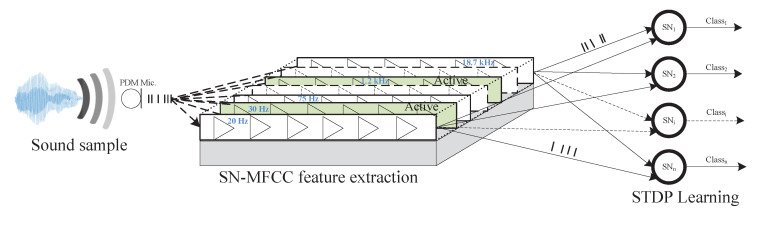
SN-MFCC based sound classification framework.

Figure 9 demonstrates the classification process which is composed of two main stages: the preprocessing phase and the classification stage. First, the SN-MFCC coefficients are extracted using the proposed SCTN-based resonator. An array of resonators is tuned to detect different frequencies in the audio spectrum, where each resonator is configured to detect a specific frequency (in the range of 20 to 20 kHz). Then, the extracted SN-MFCC features are used as an input to the SN classifier. Each SN in the output layer is independently trained, with random weight initialization, to classify one of the ten classes (selected from the RWCP database) using unsupervised learning and STDP learning rule.

**Figure 10:**
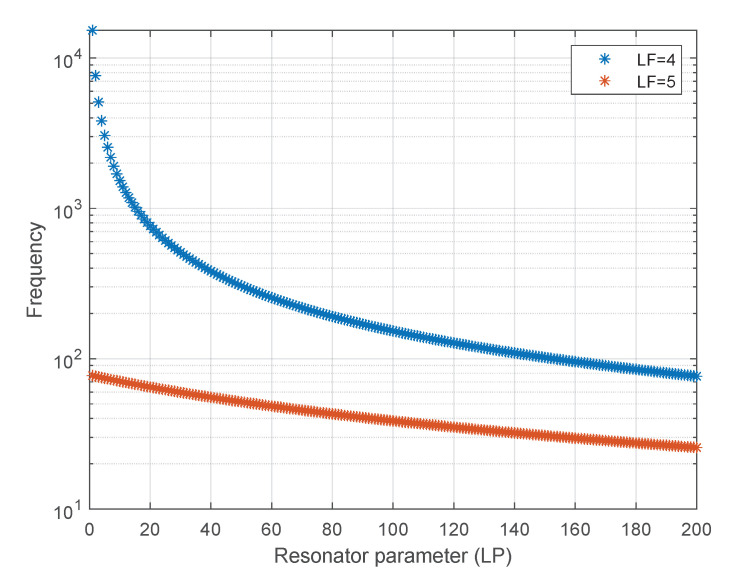
Resonators’ detection frequencies as a function of LP and LF.

The STDP module has been configured with a relatively small learning rate $\eta^{+}$ = 0.005 and $\eta^{-}$ = 0.004 to ensure robust learning [25], a time constant of $\eta^{+}$= $\eta^{-}$= 15 ms to ensure stable competitive synaptic modification [19], and the Lw parameter was set to 16. The SCTN-based resonators have been configured to detect frequencies in the range of 38 Hz to 15 kHz using varying LP and LF parameters (LF = 4:5 and LP = 1:200). Figure 10 depicts the derived frequencies as a function of the combination of the LF and LP parameters.

**Figure 11:**
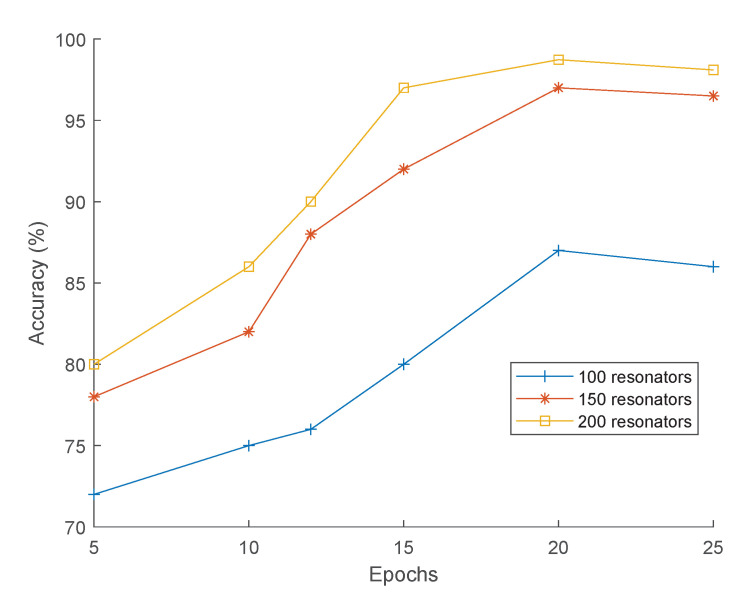
Accuracy as a function of epochs for different numbers of resonators.

The effect of the number of SN-MFCC coefficients on the classification accuracy has been investigated using 100, 150, and 200 resonators (each composed of 17 neurons). Figure 11 depicts the classification accuracy as a function of the number of SN-MFCC coefficients (for 100, 150, and 200 resonators) and the number of training epochs (for 5, 10, 12, 15, 20, and 25 epochs). An epoch refers to one cycle through the full 200 samples training dataset. Training the network with more epochs leads to better generalization and improved classification accuracy. The average test accuracy achieved with 20 epochs is 87.23%, 97.11%, and 98.73% for 100, 150, and 200 SN-MFCC coefficients, respectively.

The classification accuracy is compared with two other existing SNN-based approaches [16,26] using the same RWCP database. Table 1 depicts the accuracy results of our proposed classifier compared to RNN, LSTM [16], and three other SNN-based sound classification [16,26,38].

Simulation results of the proposed SCTN-based classifier show accuracy of 98.73%, which is comparable to the accuracy of 97–99.6% demonstrated by the SOM-SNN approach presented in [16], and outperforms the time-frequency encoding method [26], which shows the accuracy of 96%. One of the main advantages of the proposed approach is that the feature extraction is carried out using SCTN-based resonators, avoiding the common external preprocessing stage and time-frequency representation of the sound. Moreover, we suggest a direct interfacing of the acoustic sensor with the SCTN-based network avoiding the usage of costly digital-to-analog conversions.

## 6. Conclusions

This work presents a new approach based on a spiking neural network for sound preprocessing and classification. We propose a biologically plausible sound classification framework that uses an SNN-based network for detecting the embedded frequencies contained within an acoustic signal. A new SCTN digital neuron is used as a basic building block for constructing a spiking neural network. We demonstrate the use of an SCTN-based network as an efficient alternative to the design of programmable analog electronics circuits. The proposed approach is applied to sound signal processing and classification, suggesting direct interfacing of the analog sensor with the SNN network. We present an efficient ultra-low-power implementation of some common analog circuits used for phase and frequency detection, voice feature extraction, and classification. The SCTN is used as the basic building block to implement analog-like circuits, utilizing the unique features of the SCTN neuron and smart inter-connections. Experimental results show high accuracy of 98.73% achieved for sound classification. The benefit of using an SCTN-based network for implementing analog circuits is also reflected by a generic solution and the reuse ability by changing the network weights. The proposed approach can be applied in future work for efficiently extracting the common MFCC coefficients representing a speech signal.

## 7. Patents

Haiut, M. Neural cell and a neural network. Patent 15/877459, 9 August 2018.

## Figures and Tables

**Table 1:** Classification accuracy comparison (adapted and completed from the work in [16]).

Model	Accuracy (%)
RNN	95.35
LSTM	98.40
LSF-SNN	98.50
LTF-SNN	97.50
SOM-SNN	99.60
SCTN-SNN	98.73
